# Evaluation of the pharmacokinetic interactions of montmorillonite powder or loperamide on pyrotinib in healthy volunteers

**DOI:** 10.3389/fphar.2025.1563556

**Published:** 2025-05-12

**Authors:** Yike Wang, Dai Li, Tong Zhang, Sumei Xu, Yanxin Zhang, Kaijing Zhao, Shaorong Li, Kai Shen, Xiaomin Li, Pingsheng Xu

**Affiliations:** ^1^ Department of Clinical Pharmacology, Jiangsu Hengrui Pharmaceuticals Co., Ltd., Shanghai, China; ^2^ Phase I Clinical Trial Center, Xiangya Hospital, Central South University, Changsha, China; ^3^ National Clinical Research Center for Geriatric Disorders, Xiangya Hospital, Central South University, Changsha, China

**Keywords:** pyrotinib, montmorillonite powder, loperamide, diarrhea, pharmacokinetics

## Abstract

**Aims:**

To investigate the potential pharmacokinetic interactions of montmorillonite powder or loperamide on pyrotinib.

**Methods:**

This study was a single-center, open-label, single-dose, fixed-sequence clinical trial conducted with healthy volunteers. The participants were divided into two groups (A and B), each consisting of 18 subjects. Both groups received a single oral dose of 400 mg of pyrotinib on day 1. On day 9, Group A received a single dose of 400 mg of pyrotinib followed by 3 g of montmorillonite powder 2 h later, while Group B received a single dose of pyrotinib and 4 mg of loperamide after breakfast on day 9, followed by single oral doses of 2 mg of loperamide at 2 and 4 h post-administration. Blood samples were collected to determine pyrotinib blood concentrations.

**Results:**

In Group A, the combination treatment with montmorillonite powder resulted in a decrease in C_max_, AUC_0-t_, and AUC_0- ∞_ by 26.7%, 33.1%, and 32.4%, respectively, compared to pyrotinib alone. In Group B, the combination treatment with loperamide had minimal impact on pyrotinib’s absorption rate but slightly increased AUC_0-t_ and AUC_0- ∞_ by approximately 18% and 19%, respectively, while decreasing CL/F and prolonging the t_1/2_.

**Conclusion:**

Even when montmorillonite powder was administered 2 h after pyrotinib dosing, it still reduced systemic exposure of pyrotinib by 32.4% in AUC_0- ∞ ._ In contrast, loperamide increased pyrotinib exposure by 19% in AUC_0- ∞_ when used together. Based on these findings, loperamide is recommended for symptom control, while montmorillonite powder should not be co-administered with pyrotinib or any drug requiring optimal absorption.

**Clinical trial registration:**

[ClinicalTrials.gov], identifier [NCT05252546].

## Highlights

What is already known about this subject:

- Pyrotinib is an irreversible dual Human Epidermal growth factor Receptor 2(HER2) and epidermal growth factor receptor(EGFR) tyrosine kinase inhibitor(TKI). Diarrhea is the most common adverse reaction observed in clinical trials of pyrotinib.
- Symptomatic management of diarrhea of pyrotinib is usually done by using either montmorillonite powder or loperamide. The population pharmacokinetics found concomitant use of montmorillonite powder could decrease the bioavailability of pyrotinib by 50.3%.
- This study investigated the interaction of montmorillonite powder or loperamide on pyrotinib.

What this study adds:

- Even when montmorillonite powder was administered 2 hours after pyrotinib dosing, it still reduced systemic exposure of pyrotinib by 32.4% in AUC0- ∞ , potentially through a coating effect on the gastrointestinal mucosa.
- Loperamide could increase pyrotinib exposure by 19% in AUC0- ∞ potentially through reduced propulsive peristalsis and increased absorption time.
- Loperamide is recommended in symptom control in pyrotinib-induced diarrhea.

## 1 Introduction

Receptor tyrosine kinases (RTKs) are transmembrane proteins that play a crucial role in growth factor signaling transduction. To date, nearly 60 RTKs have been identified, classified into 20 distinct families. Dysregulated expression or activation of RTKs is closely associated with tumorigenesis, tumor progression, metastasis, and resistance to chemotherapy. RTK inhibitors have demonstrated efficacy in various malignancies. For instance, EGFR inhibitors, including erlotinib and gefitinib, have significantly improved outcomes in non-small cell lung cancer (NSCLC) patients harboring epidermal growth factor receptor (EGFR) mutations (Roskoski, 2023). Vascular endothelial growth factor receptor (VEGFR) inhibitors, such as sunitinib and sorafenib, have been widely used in the treatment of renal cell carcinoma and hepatocellular carcinoma by targeting tumor angiogenesis (Roskoski, 2023). Another well-characterized example is the epidermal Human Epidermal growth factor Receptor 2 (HER2), which is overexpressed in approximately 30% of human tumors (Slamon et al., 1987), including breast cancer. HER2 overexpression serves as an independent prognostic factor for recurrence and survival in breast cancer patients, making it a critical target for therapeutic intervention. As a result, multiple HER2-targeting agents, such as trastuzumab and pertuzumab, have been approved for clinical use.

Pyrotinib is an irreversible dual inhibitor of HER2 and EGFR tyrosine kinases. In China, the combination of 400 mg pyrotinib and capecitabine is approved as a second-line treatment for HER2-positive metastatic breast cancer (Yan et al., 2020; Xu et al., 2021). In healthy volunteers, the time to peak concentration following oral administration of 400 mg pyrotinib is approximately 5 h. Pyrotinib is primarily metabolized in liver by the Cytochrome P450 3A4 (CYP3A4) enzyme. The apparent volume of distribution in healthy volunteers ranges from 2,248 to 7,870 L, and the elimination half-life is approximately 18 h (data on file).

The PHILA study demonstrated that pyrotinib, when combined with trastuzumab and docetaxel, significantly improved progression-free survival (PFS) compared to placebo, trastuzumab, and docetaxel in patients with untreated HER2-positive metastatic breast cancer (24.3 versus 10.4 months; hazard ratio 0.41) (Ma et al., 2023). Diarrhea was the most common adverse event the PHILA study, occurring in nearly all patients in the pyrotinib group (293/297, 99%), with 46% (138/297) experiencing grade 3 or higher severity. Diarrhea usually lasts for 2–3 days, and the vast majority of cases could be controlled by drug withdrawal or dose reduction and symptomatic treatment (Ma et al., 2023).

Montmorillonite powder or loperamide were allowed in secondary prevention and intervention for diarrhea in PHILA study and diarrhea was generally manageable. Loperamide can bind to intestinal wall opioid receptors, inhibit acetylcholine and prostaglandin release, reduce propulsive peristalsis, and increase intestinal transit time. Loperamide is mostly absorbed by the intestinal wall, but due to the significant first-pass effect, its bioavailability is only about 0.3% (Janssen Pharmaceutica Inc., IMODIUM package insert. (2016)). The antidiarrheal gastro-intestinal protectant activity of montmorillonite powder is due to its powerful coating property on the gastrointestinal mucosa. By interacting with the glycoprotein of mucus, it increases the resistance of the mucosal gel in response to aggressive agents. Montmorillonite powder is not absorbed or metabolized (SMECTA Consumer Medication Information Leaflet, 2019).

Ad hoc analysis showed median PFS of those who had used montmorillonite powder in diarrhea management were 27.83 months, compared to 19.5 months in loperamide (data on file). However, the population pharmacokinetics of pyrotinib found concomitant use of montmorillonite could decrease the bioavailability of pyrotinib by 50.3% (Wen et al., 2021). And real-world clinical feedback indicates that patients using montmorillonite powder to manage diarrhea may experience reduced anti-tumor efficacy of pyrotinib. Given the high incidence of diarrhea during pyrotinib treatment for HER2-positive breast cancer, questions remain regarding the recommended antidiarrheal medication in the pyrotinib prescribing information. This study would evaluate the effects of montmorillonite powder and loperamide on the pharmacokinetics of pyrotinib in healthy volunteers to the inform the drug label instructions.

## 2 Methods

### 2.1 Subjects

Eligible subjects were healthy male and female volunteers aged 18–45 years, with a minimum of three subjects per sex, and a body mass index (BMI) ranging from19∼26 kg/m^2^. Participants were excluded if they exhibited abnormalities in physical examinations, laboratory profiles, vital signs, or 12-lead electrocardiograms (ECGs). Participants who required bowel motility inhibitors or had taken medications that affect liver enzyme activity within 4 weeks prior to the administration of the study drug were also excluded.

### 2.2 Treatment protocol

The study was a single-center, open-label, single-dose, fixed-sequence clinical trial conducted in healthy adult subjects (ClinicalTrials.gov identifier: NCT05252546). All research protocols received approval from the Ethics Committee of Xiangya Hospital of Central South University.

**FIGURE 1:**
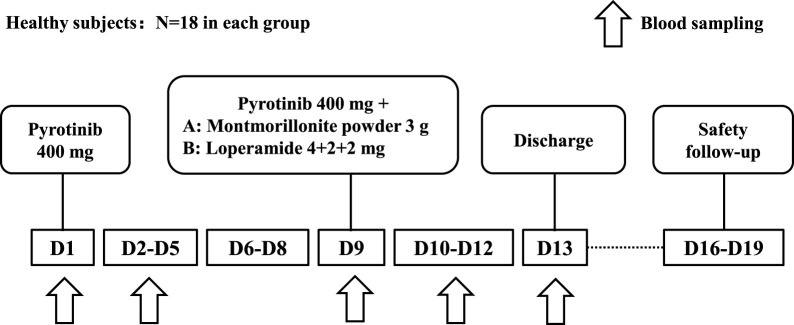
Study diagram.

As summarized in Figure 1, the participants were divided into two groups: Group A (montmorillonite powder) and Group B (loperamide), with 18 subjects in each group, resulting in a total of 36 subjects. On day 1, all subjects received a single oral dose of 400 mg pyrotinib after breakfast. On day 9, subjects in Group A received a single oral dose of 400 mg pyrotinib after breakfast, followed by a single oral dose of 3 g of montmorillonite powder 2 h later. Subjects in Group B received a single oral dose of 400 mg pyrotinib and 4 mg loperamide after breakfast on day 9, followed by additional doses of 2 mg loperamide at 2 h and 4 h post-administration. Subjects were instructed to have a light meal the night before the administration of the drug and to fast for 10 h overnight.

The timing of concomitant drug administration was designed to reflect clinically relevant conditions rather than to capture the maximum drug interaction seen in index studies (U. S. Food and Drug Administration, 2020b).

### 2.3 Blood sampling for pharmacokinetic analyses

Blood samples were collected at baseline (within 30 min prior to drug administration) and at 1, 2, 3, 4, 5, 6, 7, 8, 10, 12, 24, 48, 72, and 96 h following pyrotinib administration on days 1 and 9 to measure the concentration of pyrotinib in the plasma. After completion of the study on day 13, subjects underwent a final examination and were subsequently discharged. A safety follow-up was conducted between days 16 and 19.

The analysis of plasma samples for pyrotinib was based on a validated and sensitive liquid chromatography-tandem mass spectrometry (LC-MS/MS) method with high specificity. The plasma samples were stored at −70°C and analyzed within a stability period of 176 days. The calibration range for the standard curve was between 1.00 and 500 ng/mL, and the regression equation used linear fitting with a weighting factor of W = 1/X^2^ (bioanalysis method validation report, data on file). In each batch of analysis, the number of quality control (QC) samples accounted for more than 5% of the unknown samples in that batch. The inter-batch precision of the QC samples for low, medium, and high concentrations (LQC, 3.00 ng/mL; MQC, 25.0 ng/mL; and HQC, 400 ng/mL, respectively) was 5.9%, 6.1%, and 5.3%, respectively. The inter-batch deviation between the low, medium, and high concentration QC levels and the theoretical concentrations was −2.9%, 1.5%, and −0.5%, respectively. Representative chromatograms of pyrotinib at the LLOQ (lower limit of quantification) and in a plasma sample can be found in the Supplementary Material.

### 2.4 Statistical analysis

The individual pharmacokinetic parameters of pyrotinib would be calculated using non-compartmental analysis (NCA) and summarized by groups and combination status with descriptive statistics. To estimate the difference in the least-square means between the combination treatment and pyrotinib alone, a mixed-effects model was used to analyze the natural log-transformed pharmacokinetic parameters of pyrotinib (including AUC_0- ∞ _, AUC_0-t_, and C_max_) for each group. The model included treatment as a fixed effect and subject as a random effect. Results were exponentiated to obtain geometric least-squares means, geometric mean ratios, and corresponding 90% confidence intervals (CIs) on the original scale. Assuming a geometric mean ratio of 1.3 for the AUC of the combination treatment compared to pyrotinib alone, with an 80% acceptance range and within-subject standard deviations (log-transformed AUC) of 0.3, a sample size of 16 subjects yields a 90% confidence interval width of less than 0.223. To account for a 10% dropout rate, 18 subjects were planned for each of Groups A and B, resulting in a total of 36 subjects.

### 2.5 Safety analysis

Safety assessments included adverse events (AEs), physical examinations, vital signs, 12-lead ECGs, and clinical laboratory tests (hematology, serum chemistry, and urinalysis). All AEs were coded using the Medical Dictionary for Regulatory Activities (MedDRA^®^), version 25.0, and summarized by groups and combination status with loperamide or montmorillonite powder.

## 3 Results

### 3.1 Participants

The study was conducted from 2 March 2022 to 2 August 2022. A total of 36 subjects completed the screening assessments, met all eligibility criteria, and received the scheduled study drug. All subjects had completed each scheduled pharmacokinetic blood sample collection. One subject in each of Groups A and B was lost to follow-up during the monitoring of a grade 1 hypertriglyceridemia AE. Demographic characteristics are available for all 36 subjects, as shown in Table 1. The baseline characteristics are generally comparable between the two groups. Approximately one-fourth of the subjects were female, and the majority were of Han ethnicity.

**TABLE 1:** Demographic and baseline characteristics.

Characteristic	Group A (N = 18) (Montmorillonite powder)	Group B (N = 18) (Loperamide)	Total (N = 36)
Median age, years (range)	27.5 (22–43)	29.0 (21–38)	28.0 (21–43)
Sex, n (%)			
Male	13 (72.2%)	14 (77.8%)	27 (75.0%)
Female	5 (27.8%)	4 (22.2%)	9 (25.0%)
Ethnicity, n (%)			
Han	16 (88.9%)	17 (94.4%)	33 (91.7%)
Other	2 (11.1%)	1 (5.6%)	3 (8.3%)
Mean height, cm (SD)	166.39 (6.411)	167.92 (7.371)	167.15 (6.852)
Mean weight, kg (SD)	62.49 (5.401)	63.53 (8.179)	63.01 (6.851)
Mean BMI, kg/m^2^ (SD)	22.60 (1.941)	22.46 (1.809)	22.53 (1.851)

SD, standard deviation.

### 3.2 Pharmacokinetics

**FIGURE 2:**
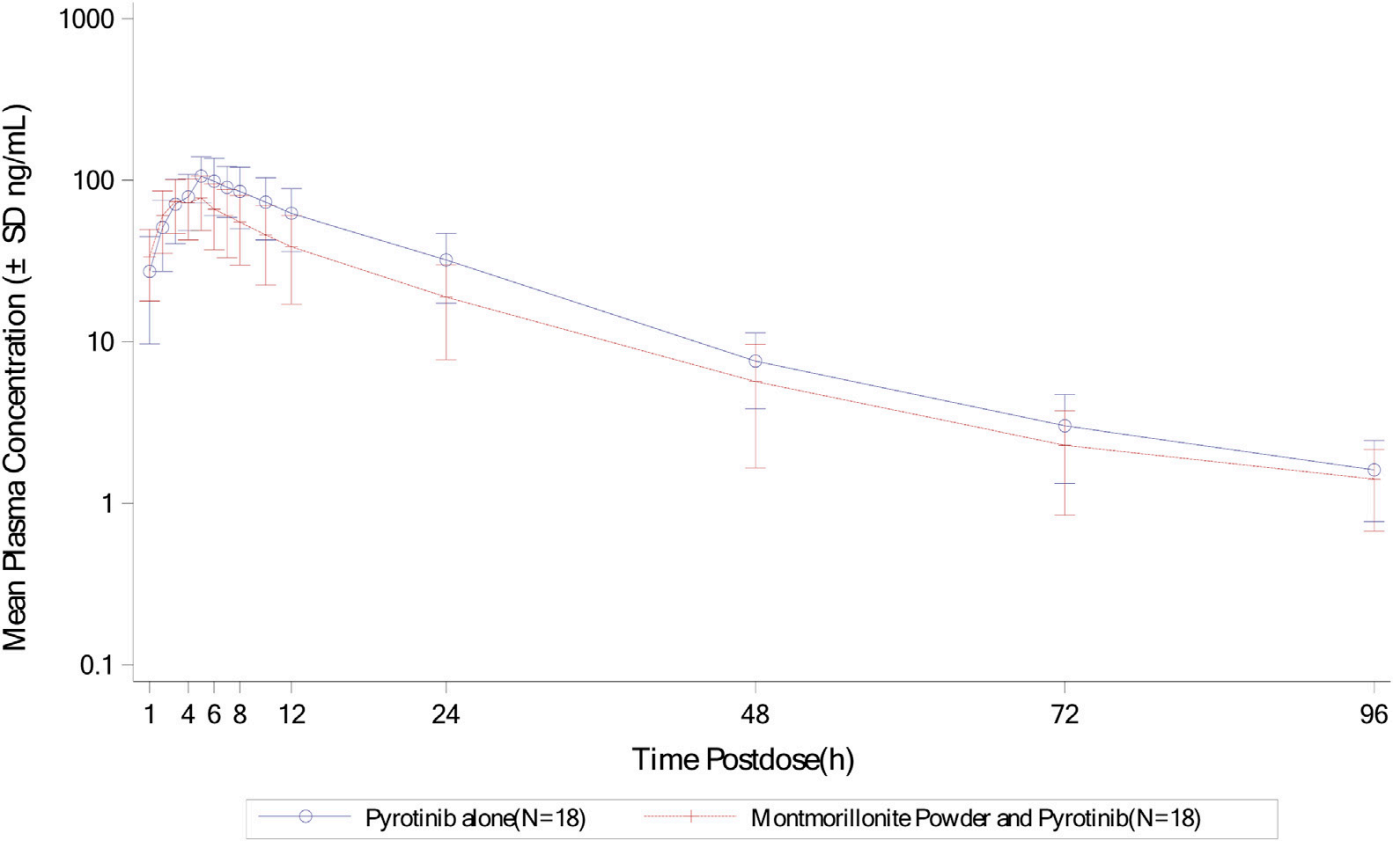
Mean plasma concentration-time profiles (semi-log) of plasma pyrotinib in Group A.

Mean plasma concentration-time profiles in semi-log scale of pyrotinib in Group A can be found in Figure 2. The pharmacokinetic parameters and statistical comparison in Group A are presented in Tables 2, 3, respectively. The geometric mean AUC_0-t_, AUC_0- ∞_ and C_max_ of plasma pyrotinib were 1835 h·ng/mL, 1877 h·ng/mL, and 105 ng/mL without montmorillonite powder, compared to 1,228 h·ng/mL, 1,268 h·ng/mL, and 77.1 ng/mL with montmorillonite powder. The median T_max_ was 5.00 h with pyrotinib alone and 3.51 h when combined with montmorillonite powder. The mean elimination t_1/2_ and geometric mean CL/F and V_z_/F were 15.5 h, 213 L/h and 4583 L with pyrotinib alone, compared to 16.3 h, 315 L/h, and 7119 L with montmorillonite powder. The mixed-effects model analysis showed that the geometric mean ratios (combination treatment/pyrotinib alone) and their 90% CIs for C_max_, AUC_0- ∞ _, and AUC_0-t_ were 0.733 (0.670, 0.803), 0.676 (0.610, 0.749), and 0.669 (0.601, 0.745), respectively. Following the combination with montmorillonite powder, the C_max_, AUC_0- ∞ _, and AUC_0-t_ of pyrotinib decreased by 26.7%, 32.4%, and 33.1%, respectively.

**TABLE 2:** Pyrotinib pharmacokinetic parameters in Group A (montmorillonite powder).

Parameters	Pyrtonib	Pyrotinib + montmorillonite powder
AUC_0-t_ (h*ng/mL)	1835 (40.6)	1,228 (48.8)
AUC_0- ∞ _(h*ng/mL)	1877 (40.5)	1,268 (48.0)
C_max_ (ng/mL)	105 (34.4)	77.1 (40.8)
CL/F (L/h)	213 (40.5)	315 (48.0)
T_max_(h)^a^	5.00 (2.98, 8.00)	3.51 (1.98, 6.00)
t_1/2_(h)^b^	15.5 (4.78)	16.3 (4.75)
V_z_/F(L)	4,583 (28.1)	7,119 (41.5)

Data are geometric mean (geometric coefficient of variation).

^a^ For T_max_, median (minimum, maximum).

^b^ For t_1/2_, mean (SD).

AUC, area under the curve; C_max_, peak plasma concentration; T_max_, time to peak plasma concentration; t_1/2_, elimination half-life; CL/F, apparent clearance; V_z_/F, apparent volume of distribution.

**TABLE 3:** Statistical comparison of pyrotinib plasma pharmacokinetic parameters for pyrotinib in combination with montmorillonite powder vs. pyrotinib alone.

Pharmacokinetic parameters	Geometric least-square mean*		% Ratio of least-square means (90% CI)
	Pyrotinib alone	Pyrotinib + montmorillonite powder	
C_max_ (ng/mL)	105	77.1	73.3 (67.0,80.3)
AUC_0- ∞ _ (h*ng/mL)	1880	1,270	67.6 (61.0,74.9)
AUC_0-t_ (h*ng/mL)	1830	1,230	66.9 (60.1,74.5)

*Values were reported with three significant figures.

**FIGURE 3:**
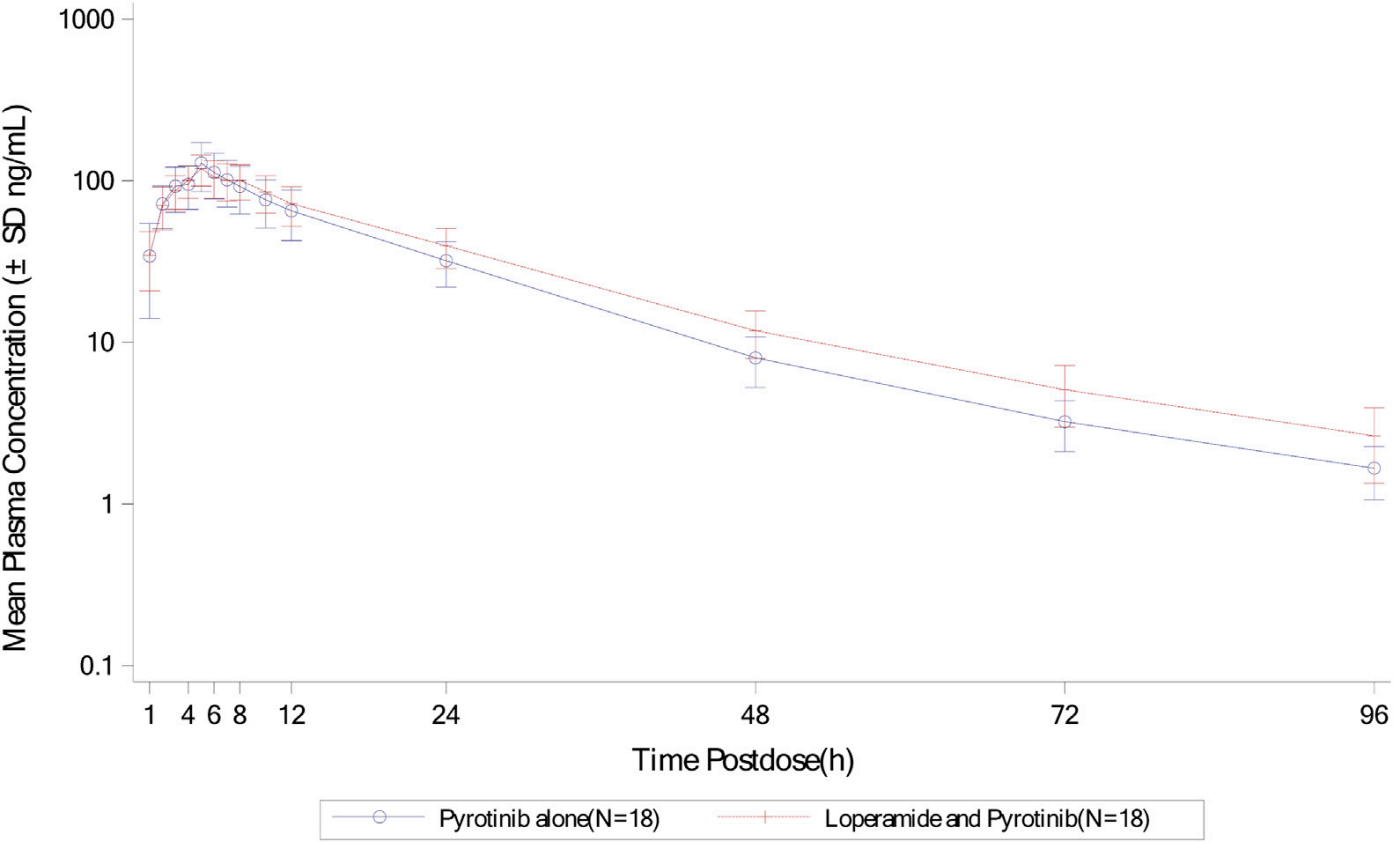
Mean plasma concentration-time profiles (semi-log) of plasma pyrotinib in Group B.

Mean plasma concentration-time profiles in semi-log scale of pyrotinib in Group B can be found in Figure 3. The pharmacokinetic parameters and statistical comparison in Group B are presented in Tables 4, 5. The geometric mean AUC_0-t_, AUC_0- ∞_ and C_max_ of plasma pyrotinib were 2065 h*ng/mL, 2,112 h*ng/mL and 124 ng/mL without loperamide, compared to 2,427 h*ng/mL, 2,505 h*ng/mL, and 118 ng/mL with loperamide. The mean elimination half-life (t_1/2_) and geometric mean CL/F and V_z_/F was 17.3 h, 189 L/h and 4525 L with pyrotinib alone, compared to 19.9 h, 160 L/h, and 4491 L with loperamide. The mixed-effects model analysis showed that the geometric mean ratio (combination treatment/pyrotinib alone) and its 90% confidence interval for C_max_, AUC_0- ∞ _, and AUC_0-t_ were 0.946 (0.867, 1.03), 1.19 (1.09, 1.29), and 1.18 (1.08, 1.28), respectively. Following the combination of loperamide with pyrotinib, the C_max_ of pyrotinib decreased by approximately 5.4%, while the AUC_0- ∞_ and AUC_0-t_ increased by approximately 19% and 18%, respectively.

**TABLE 4:** Pyrotinib pharmacokinetic parameters in Group B (loperamide).

Parameters	Pyrtonib	Pyrotinib + loperamide
AUC_0-t_ (h*ng/mL)	2,065 (27.3)	2,427 (24.2)
AUC_0- ∞ _(h*ng/mL)	2,112 (26.7)	2,505 (24.5)
C_max_ (ng/mL)	124 (33.4)	118 (20.7)
CL/F (L/h)	189 (26.7)	160 (24.6)
T_max_(h)^a^	4.98 (2.00, 5.00)	5.00 (1.98, 6.03)
t_1/2_(h)^b^	17.3 (5.18)	19.9 (4.29)
V_z_/F(L)	4,525 (38.6)	4,491 (24.5)

Data are geometric mean (geometric coefficient of variation).

^a^ For T_max_, median (minimum, maximum).

^b^ For t_1/2_, mean (SD).

AUC, area under the curve; C_max_, peak plasma concentration; T_max_, time to peak plasma concentration; t_1/2_, elimination half-life; CL/F, apparent clearance; V_z_/F, apparent volume of distribution.

**TABLE 5:** Statistical comparison of pyrotinib plasma pharmacokinetic parameters for pyrotinib in combination with loperamide vs. pyrotinib alone.

Pharmacokinetic parameters	Geometric least-square mean*		% Ratio of least-square means (90% CI)
	Pyrotinib alone	Pyrotinib + loperamide	
C_max_ (ng/mL)	124	118	94.6 (86.7, 103)
AUC_0- ∞ _ (h*ng/mL)	2,110	2,510	119 (109, 129)
AUC_0-t_ (h*ng/mL)	2060	2,430	118 (108, 128)

*Values were reported with three significant figures.

Overall, the results of Group A showed that there was no significant change in t_1/2_ of pyrotinib when used in combination with montmorillonite powder compared to pyrotinib alone. Median time to maximum pyrotinib concentration was 3.51 h when combined with montmorillonite powder, which was earlier than the 5 h observed in the single treatment group. The results of Group B showed that after combination with loperamide, there was no significant change in the median T_max_ compared to pyrotinib alone. The exposure of pyrotinib (C_max_) was similar, while CL/F decreased which may be explained by increased F mathematically or reduced propulsive peristalsis and increased absorption time mechanistically. Loperamide had minimal impact on the absorption rate of pyrotinib, with a decrease in C_max_ of approximately 5.4%, and an increase in AUC_0-t_ and AUC_0- ∞_ by approximately 18% and 19%, respectively, indicating a mild influence of loperamide on the pharmacokinetics of pyrotinib.

### 3.3 Safety results

A total of eight subjects in Group A experienced 11 treatment-emergent adverse events (TEAEs), all of which were mild (Grade 1) in severity and occurred during the treatment period. Common TEAEs (experienced by ≥ 2 subjects) included hypertriglyceridemia (11.1%). Other TEAEs included hypercholesterolemia, nausea, abdominal distension, alanine aminotransferase elevation, aspartate aminotransferase elevation, bradycardia, elevated blood bilirubin, upper respiratory tract infection, and orthostatic hypotension (each occurring in 5.6% of subjects).

In Group B, a total of 12 subjects experienced 18 TEAEs. Except for one subject who experienced a Grade 2 hypertriglyceridemia event, and another subject who experienced a Grade 2 rash event, all other TEAEs were mild (Grade 1) in severity. Common TEAEs (experienced by ≥ 2 subjects) included hypertriglyceridemia (22.2%), hyperuricemia (11.1%), oral ulcer (11.1%), and rash (11.1%). Other TEAEs included nausea, abdominal distension, PR interval prolongation on electrocardiogram, increased heart rate, increased total bile acid, headache, and dizziness (each occurring in 5.6% of subjects).

No TEAEs resulted in subject withdrawal, treatment discontinuation, or death in either Group A or Group B. No serious adverse events were reported. Overall, the safety profile of a single oral dose of pyrotinib alone or in combination with montmorillonite powder or loperamide was manageable in healthy subjects.

## 4 Discussion

The guidance recommended that doses of the perpetrator drug in DDI studies should maximize the possibility of identifying a DDI (U. S. Food and Drug Administration, 2020a; U. S. Food and Drug Administration, 2020b). However, for concomitant-use studies, a risk-based approach to evaluate the interaction in clinical settings should be adopted (U. S. Food and Drug Administration, 2020b). The timing of drug administration should be based on the purpose. The guidance recommended that the sponsor should adjust the timing of drug administration to maximize the potential to detect an interaction in index studies) and/or to reflect the clinically relevant conditions in concomitant-use studies (U. S. Food and Drug Administration, 2020b).

The clinically relevant conditions for concomitant drugs are from clinical trial or guidance. An average of three sachets per day is recommended for montmorillonite powder in the label. The interval between administration of montmorillonite powder and pyrotinib is at least 2 h in clinical trials. Therefore, the administration method used in this protocol is to administer montmorillonite powder 2 h after pyrotinib administration. According to the “Expert consensus on the management of adverse events of ErbB family tyrosine kinase inhibitors (TKI) in breast cancer”, diarrhea caused by small-molecule TKIs targeting the HER2 receptor should be treated with an initial dose of 4 mg of loperamide, followed by 2 mg every 2 h, up to a maximum of 16 mg (Wang et al., 2020). To reduce the risk of constipation in healthy volunteers, the maximum daily dose of loperamide is restricted to 8 mg. The regimen of loperamide in the study is to administer 4 mg in combination with pyrotinib, followed by 2 mg of loperamide 2 and 4 h after pyrotinib administration.

Loperamide works by slowing down the movement of the intestines and can increase the residence time of pyrotinib (Janssen Pharmaceutica Inc., 2016). This extended residence time allows more time for pyrotinib to be absorbed, which is expected to significantly increase the AUC, reflecting the total drug exposure over time. In contrast, the maximum concentration may not be as significantly affected, as it is influenced by the rate of absorption, which can be less impacted by slowed motility than total absorption over time. Thus, a greater change in AUC is anticipated compared to C_max_, as the overall exposure to pyrotinib is prolonged, while the peak concentration might remain similar or change less markedly. This hypothesis is supported by the study results, which showed a more pronounced effect on AUC than on C_max_. Though not included in the package insert, literature has reported that loperamide was a potent inhibitor of CYP3A4 with an IC_50_ of 0.050 µM (Marechal et al., 2006). Interaction potential should be evaluated since pyrotinib is mainly metabolized through the CYP3A4 pathway. However, only 0.3% of orally administered loperamide is available in blood and 95% of them bind to albumin. The maximum concentration of loperamide after 4 mg oral administration was 0.62 ng/mL (Niemi et al., 2006). The calculated R_1_ for reversible inhibition would be less than 1.0001. Based on the available data, significant CYP3A4 inhibition by loperamide *in vivo* is unlikely at clinically relevant doses due to its low systemic bioavailability. However, the calculated R_1,gut_ for loperamide is 1,342.7 based on molecular weight of 477 Dalton, which is significantly higher than the cut off of 11 from guidance. Loperamide use may need to be limited for medications that undergoes high gut CYP3A4 metabolism.

Montmorillonite powder is a type of clay mineral that belongs to the smectite group. It brings a powerful coating on the gastrointestinal mucosa and increases the resistance of the mucosal gel in response to aggressive agents (SMECTA Consumer Medication Information Leaflet, 2019). The dedicated concomitant-use study showed good consistence to the model result. The current study further verifies that montmorillonite powder influences the absorption rate and extent of pyrotinib with 1.5 h earlier in time to maximum concentration and 32.4% lower of AUC_0- ∞ _. Montmorillonite powder was administered 2 h after pyrotinib, raising the question of whether a significant portion of pyrotinib had already been absorbed by that time. While a 2-h interval is used in clinical trials, this does not necessarily reflect real-world practice, where outpatient adherence to such timing is uncertain. Real-world clinical feedback in China has indicated that patients receiving pyrotinib with concomitant use of montmorillonite powder for diarrhea management may experience reduced anti-tumor efficacy. This observation aligns with population pharmacokinetic analyses showing that co-administration of montmorillonite reduces pyrotinib bioavailability by approximately 50.3%. Given that the 2-h interval between the two agents was adopted from the phase III clinical trial, two potential study strategies were considered. The first approach involved two separate studies: one to characterize the maximum drug–drug interaction (DDI) and support a clear warning against co-administration, and another to determine a practical dosing interval, guided by FDA food effect principles recommending interval studies when food adversely affects a drug’s efficacy or safety (U. S. Food and Drug Administration, 2022). However, this was challenged as less feasible for label recommendation. The second option, adopted in the current study, was a single study focused on evaluating the effectiveness of the commonly used 2-h interval, based on the expectation of a significant drug interaction when used at the same time. Results demonstrated a 33% reduction in exposure even with this spacing, suggesting that a significant portion of pyrotinib is still not absorbed. Rather than exploring the theoretical maximum DDI, this approach prioritized validating whether the clinically relevant interval used in practice is adequate for label recommendations. Ultimately, the data suggest that montmorillonite powder should not be co-administered with pyrotinib or any drug requiring full absorption.

## 5 Conclusion

Even when montmorillonite powder was administered 2 h after pyrotinib dosing, it still reduced systemic exposure of pyrotinib by 32.4% in AUC_0- ∞ _. In contrast, loperamide increased pyrotinib exposure by 19% in AUC_0- ∞_ when used together. Based on these findings, loperamide is recommended for symptom control, while montmorillonite powder should not be co-administered with pyrotinib or any drug requiring optimal absorption.

## Data Availability

The original contributions presented in the study are included in the article/Supplementary Material, further inquiries can be directed to the corresponding authors.
