# Supplementary material for: Evaluation of the pharmacokinetic interactions of montmorillonite powder or loperamide on pyrotinib in healthy volunteers
Source: Front Pharmacol. 2025 May 12;16:1563556. doi: 10.3389/fphar.2025.1563556 (PMC12104674; doi:10.3389/fphar.2025.1563556)

Sample Name: "5 023 HR-BLTN-2019-HPK_MV_Human Plasma LLOQ 2 1" Sample ID: "23" File: "001.wiff"
Peak Name: "BLTN" Mass(es): "583.400/138.200 Da"
Comment: "none" Annotation: ""

Sample Index: 23
Sample Type: QC
Concentration: 1.00 ng/mL
Calculated Conc: 1.00 ng/mL
Acq. Date: 2019/7/16
Acq. Time: 11:41:55

Modified: No
Proc. Algorithm: Analyst Classic
Bunching Factor: 1
Noise Threshold: 10.00 cps
Area Threshold: 50.00 cps
,Num. Smooths: 3
Sep. Width: 0.20
Sep. Height: 1.00
Exp. Peak Ratio: 5.00
Exp. Adj. Ratio: 4.00
Exp. Val. Ratio: 3.00
RT Window: 20.0 sec
Expected RT: 1.43 min
Use Relative RT: No

Int. Type: Base To Base
Retention Time: 1.428 min
Area: 4.060210154e+003 counts
Height: 2.90e+003 cps
Start Time: 1.39 min
End Time: 1.48 min

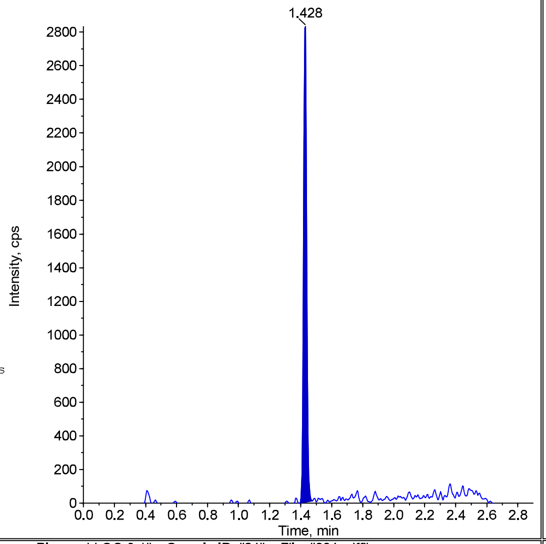

Sample Name: "5 023 HR-BLTN-2019-HPK_MV_Human Plasma LLOQ 2 1" Sample ID: "23" File: "001.wiff"
Peak Name: "IS(IS)" Mass(es): "329.200/161.900 Da"
Comment: "none" Annotation: ""

Sample Index: 23
Sample Type: QC
Concentration: 1.00 ng/mL
Calculated Conc: N/A
Acq. Date: 2019/7/16
Acq. Time: 11:41:55

Modified: No
Proc. Algorithm: Analyst Classic
Bunching Factor: 1
Noise Threshold: 50.00 cps
Area Threshold: 50.00 cps
,Num. Smooths: 3
Sep. Width: 0.20
Sep. Height: 1.00
Exp. Peak Ratio: 5.00
Exp. Adj. Ratio: 4.00
Exp. Val. Ratio: 3.00
RT Window: 20.0 sec
Expected RT: 1.39 min
Use Relative RT: No

Int. Type: Base To Base
Retention Time: 1.378 min
Area: 1.895387672e+005 counts
Height: 1.38e+005 cps
Start Time: 1.34 min
End Time: 1.44 min

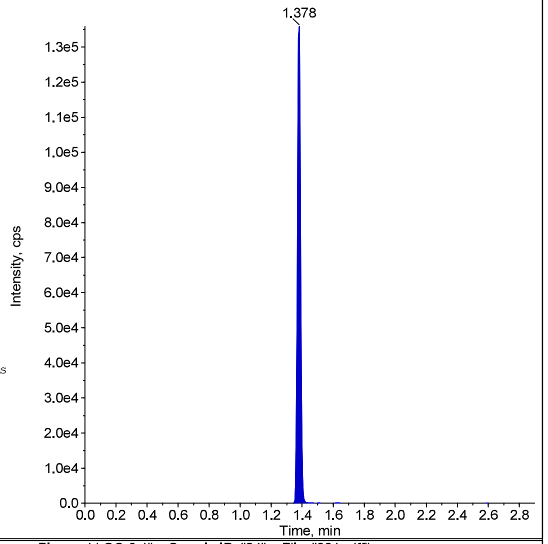

Sample Name: "5 024 HR-BLTN-2019-HPK_MV_Human Plasma LLOQ 3 1" Sample ID: "24" File: "001.wiff"
Peak Name: "BLTN" Mass(es): "583.400/138.200 Da"
Comment: "none" Annotation: ""

Sample Index: 24
Sample Type: QC
Concentration: 1.00 ng/mL
Calculated Conc: 0.953 ng/mL
Acq. Date: 2019/7/16
Acq. Time: 11:45:20

Modified: No
Proc. Algorithm: Analyst Classic
Bunching Factor: 1
Noise Threshold: 10.00 cps
Area Threshold: 50.00 cps
,Num. Smooths: 3
Sep. Width: 0.20
Sep. Height: 1.00
Exp. Peak Ratio: 5.00
Exp. Adj. Ratio: 4.00
Exp. Val. Ratio: 3.00
RT Window: 20.0 sec
Expected RT: 1.43 min
Use Relative RT: No

Int. Type: Base To Base
Retention Time: 1.428 min
Area: 3.876127634e+003 counts
Height: 2.68e+003 cps
Start Time: 1.40 min
End Time: 1.47 min

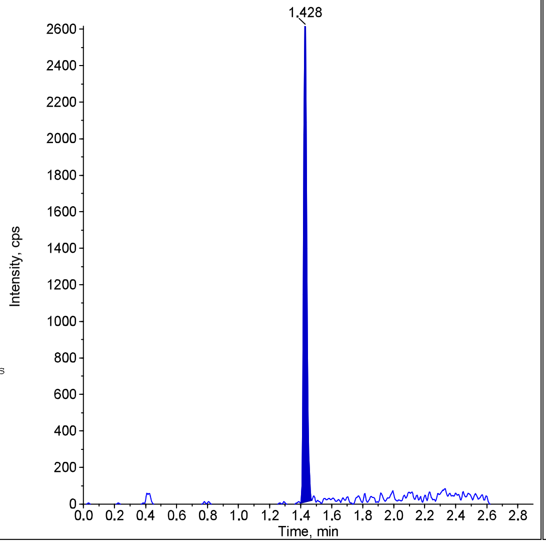

Sample Name: "5 024 HR-BLTN-2019-HPK_MV_Human Plasma LLOQ 3 1" Sample ID: "24" File: "001.wiff"
Peak Name: "IS(IS)" Mass(es): "329.200/161.900 Da"
Comment: "none" Annotation: ""

Sample Index: 24
Sample Type: QC
Concentration: 1.00 ng/mL
Calculated Conc: N/A
Acq. Date: 2019/7/16
Acq. Time: 11:45:20

Modified: No
Proc. Algorithm: Analyst Classic
Bunching Factor: 1
Noise Threshold: 50.00 cps
Area Threshold: 50.00 cps
,Num. Smooths: 3
Sep. Width: 0.20
Sep. Height: 1.00
Exp. Peak Ratio: 5.00
Exp. Adj. Ratio: 4.00
Exp. Val. Ratio: 3.00
RT Window: 20.0 sec
Expected RT: 1.39 min
Use Relative RT: No

Int. Type: Base To Base
Retention Time: 1.380 min
Area: 1.889076513e+005 counts
Height: 1.33e+005 cps
Start Time: 1.35 min
End Time: 1.44 min

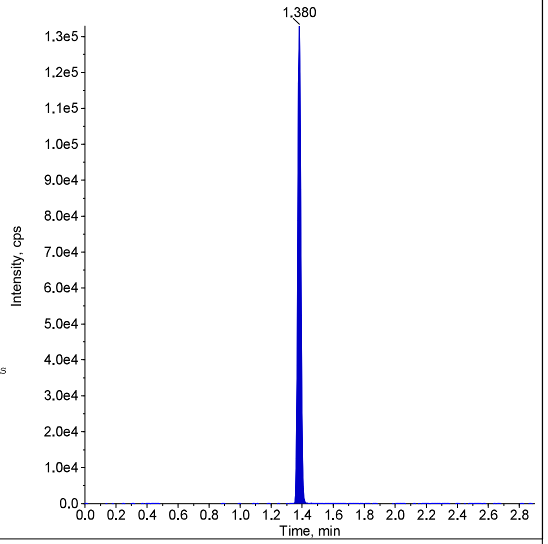

Supplement: Supplementary file 1 [file Supplementaryfile1.pdf]
