# Supplementary material for: Evaluation of the pharmacokinetic interactions of montmorillonite powder or loperamide on pyrotinib in healthy volunteers
Source: Front Pharmacol. 2025 May 12;16:1563556. doi: 10.3389/fphar.2025.1563556 (PMC12104674; doi:10.3389/fphar.2025.1563556)

Sample Name: "1 001 HR-BLTN-DDI-07 System 1" Sample ID: "1" File: "001.wiff"
Peak Name: "BLTN" Mass(es): "583.400/138.200 Da"
Comment: "none" Annotation: ""

Sample Index: 2
Sample Type: Solvent
Concentration: 0.00 ng/mL
Calculated Conc: N/A
Acq. Date: 2022/6/22
Acq. Time: 13:30:12

Modified: No
Proc. Algorithm: Analyst Classic
Bunching Factor: 1
Noise Threshold: 30.00 cps
Area Threshold: 50.00 cps
,Num. Smooths: 3
Sep. Width: 0.20
Sep. Height: 1.00
Exp. Peak Ratio: 5.00
Exp. Adj. Ratio: 4.00
Exp. Val. Ratio: 3.00
RT Window: 20.0 sec
Expected RT: 1.47 min
Use Relative RT: No

Int. Type: Base To Base
Retention Time: 1.472 min
Area: 2.594825264e+004 counts
Height: 1.71e+004 cps
Start Time: 1.42 min
End Time: 1.55 min

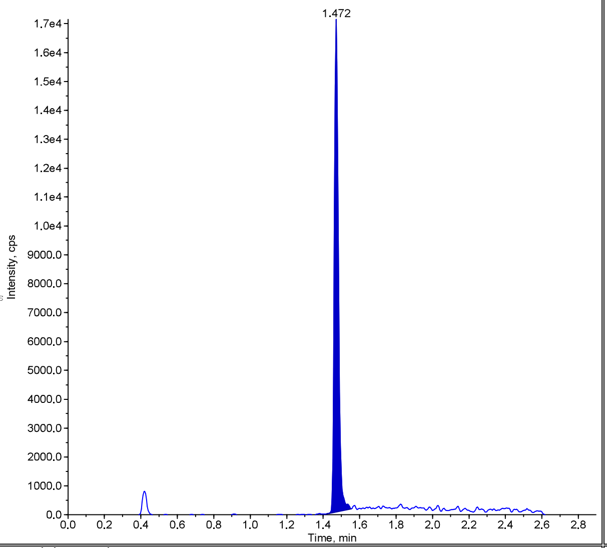

Sample Name: "1 001 HR-BLTN-DDI-07 System 1" Sample ID: "1" File: "001.wiff"
Peak Name: "IS(IS)" Mass(es): "329.200/161.900 Da"
Comment: "none" Annotation: ""

Sample Index: 2
Sample Type: Solvent
Concentration: 1.00 ng/mL
Calculated Conc: N/A
Acq. Date: 2022/6/22
Acq. Time: 13:30:12

Modified: No
Proc. Algorithm: Analyst Classic
Bunching Factor: 1
Noise Threshold: 50.00 cps
Area Threshold: 50.00 cps
,Num. Smooths: 3
Sep. Width: 0.20
Sep. Height: 1.00
Exp. Peak Ratio: 5.00
Exp. Adj. Ratio: 4.00
Exp. Val. Ratio: 3.00
RT Window: 20.0 sec
Expected RT: 1.42 min
Use Relative RT: No

Int. Type: Base To Base
Retention Time: 1.420 min
Area: 2.012922864e+005 counts
Height: 1.37e+005 cps
Start Time: 1.38 min
End Time: 1.49 min

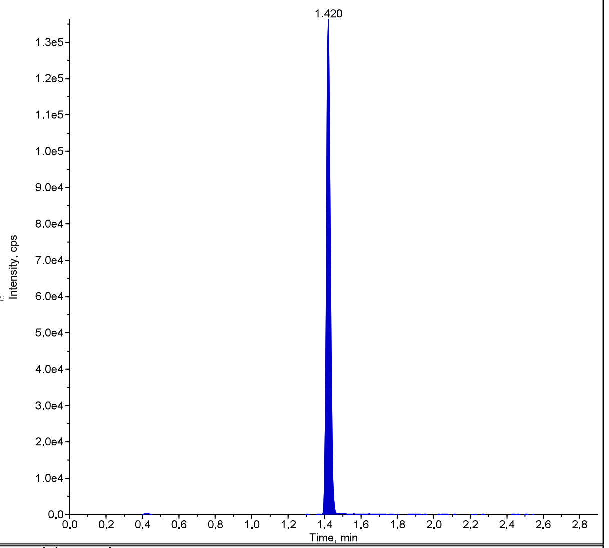

Sample Name: "1 002 HR-BLTN-DDI-07 System 1" Sample ID: "2" File: "001.wiff"
Peak Name: "BLTN" Mass(es): "583.400/138.200 Da"
Comment: "none" Annotation: ""

Sample Index: 3
Sample Type: Solvent
Concentration: 0.00 ng/mL
Calculated Conc: N/A
Acq. Date: 2022/6/22
Acq. Time: 13:33:42

Modified: No
Proc. Algorithm: Analyst Classic
Bunching Factor: 1
Noise Threshold: 30.00 cps
Area Threshold: 50.00 cps
,Num. Smooths: 3
Sep. Width: 0.20
Sep. Height: 1.00
Exp. Peak Ratio: 5.00
Exp. Adj. Ratio: 4.00
Exp. Val. Ratio: 3.00
RT Window: 20.0 sec
Expected RT: 1.47 min
Use Relative RT: No

Int. Type: Base To Base
Retention Time: 1.473 min
Area: 2.313673175e+004 counts
Height: 1.57e+004 cps
Start Time: 1.44 min
End Time: 1.52 min

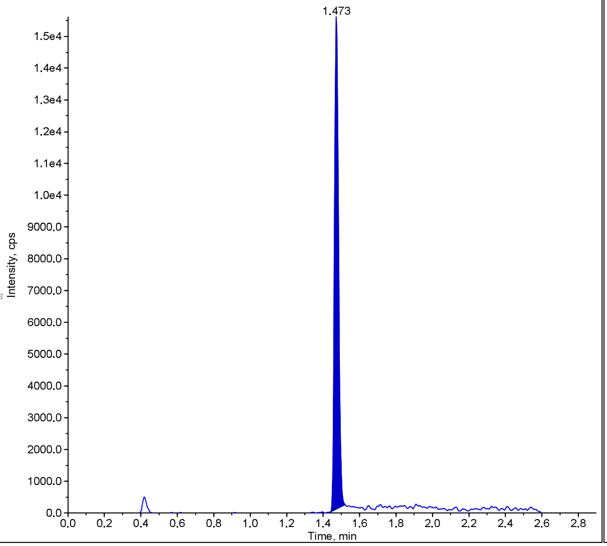

Sample Name: "1 002 HR-BLTN-DDI-07 System 1" Sample ID: "2" File: "001.wiff"
Peak Name: "IS(IS)" Mass(es): "329.200/161.900 Da"
Comment: "none" Annotation: ""

Sample Index: 3
Sample Type: Solvent
Concentration: 1.00 ng/mL
Calculated Conc: N/A
Acq. Date: 2022/6/22
Acq. Time: 13:33:42

Modified: No
Proc. Algorithm: Analyst Classic
Bunching Factor: 1
Noise Threshold: 50.00 cps
Area Threshold: 50.00 cps
,Num. Smooths: 3
Sep. Width: 0.20
Sep. Height: 1.00
Exp. Peak Ratio: 5.00
Exp. Adj. Ratio: 4.00
Exp. Val. Ratio: 3.00
RT Window: 20.0 sec
Expected RT: 1.42 min
Use Relative RT: No

Int. Type: Base To Base
Retention Time: 1.417 min
Area: 2.078661559e+005 counts
Height: 1.38e+005 cps
Start Time: 1.38 min
End Time: 1.49 min

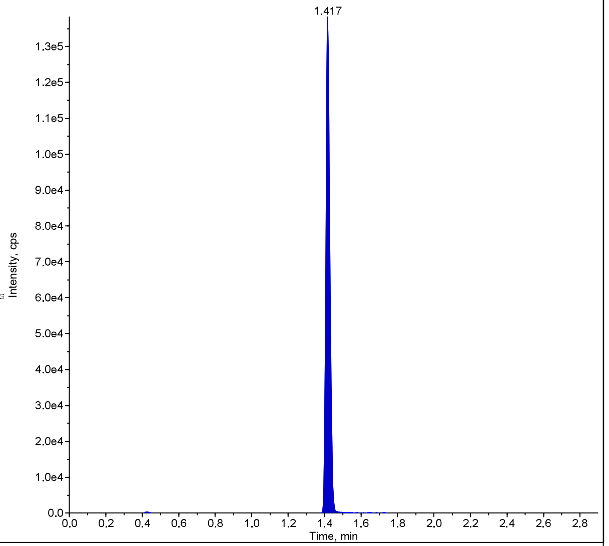

Supplement: Supplementary file 2 [file Supplementaryfile2.pdf]
